# Honokiol Is More Potent than Magnolol in Reducing Head and Neck Cancer Cell Growth

**DOI:** 10.3390/cimb46100637

**Published:** 2024-09-25

**Authors:** Robert Kleszcz, Dawid Dorna, Maciej Stawny, Jarosław Paluszczak

**Affiliations:** 1 Department of Pharmaceutical Biochemistry, Poznan University of Medical Sciences, 60-806 Poznań, Poland; kleszcz@ump.edu.pl; 2 Department of Pharmaceutical Biochemistry, Doctoral School, Poznan University of Medical Sciences, 60-806 Poznań, Poland; dawid.dorna97@gmail.com; 3 Department of Pharmaceutical Chemistry, Poznan University of Medical Sciences, 60-806 Poznań, Poland; mstawny@ump.edu.pl

**Keywords:** honokiol, magnolol, cisplatin, head and neck cancer, cisplatin persister cells, spheroids

## Abstract

The efficacy of treatment of head and neck squamous cell carcinoma (HNSCC) patients is still unsatisfactory, and there is an ongoing search for novel therapies. Locoregionally advanced HNSCC cases, which frequently require combined surgery and chemoradiotherapy, are especially difficult to treat. Natural compounds, like *Magnolia*-derived lignans—honokiol (HON) and magnolol (MAG)—can reduce cancer cell growth but retain a good safety profile and thus may show benefit as adjuvant therapeutics. The aim of this study was to evaluate the anti-cancer effects of HON and MAG in HNSCC cell lines and compare their effects between cisplatin-sensitive and cisplatin-tolerant cells. Cell viability was evaluated in FaDu and SCC-040 cells growing as monolayers and as spheroids. The effect of HON and MAG on the cell cycle, apoptosis, and gene expression was compared between wild-type FaDu cells and cisplatin persister FaDu cells. We observed that HON and MAG were more potent in reducing cell viability in cisplatin persister FaDu cells, although this effect was not directly followed by increased rates of apoptosis. Thus, HON’s and MAG’s capacity to affect cisplatin persister cells needs further studies. In general, we observed that HON exerted stronger cytotoxic effects than MAG in HNSCC cells, and the difference in their anti-cancer activity was especially pronounced in cells cultured in 3D.

## 1. Introduction

Cancer is the second leading cause of death worldwide, and head and neck squamous cell carcinoma (HNSCC) is fatal for more than 400,000 people per year [1]. Primary surgery treatment turns out to be insufficient or even impossible in a considerable number of patients; thus, it must be supported by chemo- and/or radiotherapy. Standard chemotherapeutics used to treat HNSCC are represented by cisplatin, 5-fluorouracil, and docetaxel, although currently targeted therapy regimens are also introduced, namely monoclonal antibodies against epithelial growth factor receptor (EGFR)—cetuximab, or programmed death receptor 1 (PD-1)—nivolumab and pembrolizumab [2]. For instance, to reach an overall survival rate above 50% for locally advanced HNSCC, concurrent chemoradiotherapy should be combined with targeted therapy or induction chemotherapy [3]. Approximately half of the patients recur and are single chemotherapy-resistant [4]. Thus, new concepts of adjuvant therapy for HNSCC are necessary to achieve progress in the overall survival time of HNSCC patients. Importantly, natural products are a rich source of biologically active compounds with anti-cancer activities [5], and their use provides possible benefits for patients, which requires further validation and confirmation.

Honokiol (HON), IUPAC Name 2-(4-hydroxy-3-prop-2-enylphenyl)-4-prop-2-enylphenol [6], is a lignan isolated from the bark, seed cones, and leaves of the *Magnolia officinalis* tree, being a traditional Chinese bioactive compound [7,8]. HON presents pleiotropic pharmacological activities, including antioxidant, anti-inflammatory, neuro-, hepato-, and cardio-protective, or anti-microbial effects [7,8,9,10,11,12,13]. What is essential is that the anti-cancer properties of HON against breast, colon, liver, lung, and ovarian cancer or glioblastoma were also observed [7,8,13,14,15].

**Figure 1:**
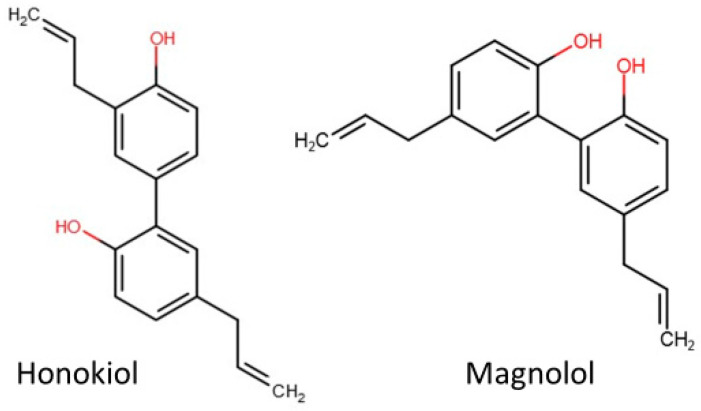
The chemical structure of honokiol and magnolol. Source: www.pol-aura.pl, accessed on 21 August 2024.

Magnolol (MAG), IUPAC Name 2-(2-hydroxy-5-prop-2-enylphenyl)-4-prop-2-enylphenol [16], is a structural isomer of HON (Figure 1), also isolated from *Magnolia officinalis*. Similarly to HON, MAG also exerts pleiotropic pharmacological effects, including anti-cancer effects [17,18,19]. However, some slight activity disparity can be observed, e.g., the difference in the position of one hydroxyl group is the cause of weaker antioxidant properties of MAG due to the formation of intramolecular hydrogen bonds between *ortho*-hydroxyl groups, which prevents the hydrogen atom from being abstracted by radicals [20,21]. This small structural difference between HON and MAG results in variation in their activities in various types of cancer cells [21].

The induction of apoptosis is a frequent consequence of effective anti-cancer therapy. HON and MAG were reported to activate the extrinsic (death receptor-mediated) and intrinsic (mitochondria-mediated) pathways of apoptotic cell death via similar molecular mechanisms [22]. However, comparable concentrations of HON and MAG provoke unequal effects on cancer cell viability and apoptosis. For instance, HON is a much stronger viability reducer in glioblastoma [23] and bladder [24] cancer cells compared to MAG. In turn, the pro-apoptotic effect of HON was more potent than MAG in glioblastoma cancer cells, while both HON and MAG used alone were insufficient to induce apoptosis in the second case [23,24].

The data describing the effects of HON and MAG in head and neck carcinoma cells are limited. Most reports concern the anti-cancer properties of HON or the effect of *Magnolia* extracts containing HON and MAG [25,26,27]. A direct comparison of these properties between HON and MAG in HNSCC cells is lacking. In addition, resistance to standard therapy represents a significant problem of HNSCC therapy. HON combined with 5-fluorouracil (5-FU) synergistically improved the anti-cancer effect in HSC-3 and HSC-4 oral cancer cell lines [28]. Based on a model of acquired resistance to cetuximab, HON also improved the efficacy of this EGFR receptor-targeted treatment [29]. MAG increased chemosensitivity to cisplatin in oral cancer cells by affecting interleukin 6 (IL-6) and STAT3 [30]. Thus, it is also interesting to evaluate the effect of HON and MAG in cisplatin-resistant HNSCC cells.

To fill the information gap mentioned above, this study aimed to compare the effects of HON and MAG on the viability of HNSCC cells grown as monolayer or 3D spheroids. Moreover, in order to evaluate the possible effects of HON and MAG in resistant HNSCC cells, we compared their effects in wild-type (wt) and cisplatin persister FaDu cells. 

## 2. Materials and Methods

### 2.1. Chemicals and Cell Culture Conditions

Honokiol and magnolol were purchased from Pol-Aura (Olsztyn, Poland), and stock solutions (20 mM) were prepared in DMSO and stored in aliquots at −20 °C. The experiments were performed using two commercially available HNSCC cell lines: FaDu hypopharyngeal cancer cells (American Type Culture Collection, ATCC, Manassas, VA, USA) and SCC-040 tongue cancer cells (German Collection of Microorganisms and Cell Cultures, DSMZ, Braunschweig, Germany). As previously described [31], the cells were grown using high-glucose Dulbecco’s Modified Eagle’s Medium (DMEM, Biowest, Nuaillé, France) supplemented with 5% Fetal Bovine Serum (FBS, EURx, Gdańsk, Poland), and 1% antibiotic solution (penicillin and streptomycin; Biowest, Nuaillé, France) under standard conditions (37 °C, 5% CO_2_, 90% humidity) in a Memmert CO_2_ incubator (Schwabach, Germany).

To generate cisplatin persister FaDu cells, a stepwise treatment regimen was applied. Initially, the wt FaDu cells were exposed to three doses of cisplatin at the IC25 concentration (1.12 µM, established during our previous study [31]), with each dose administered three days apart. Following this initial treatment phase, the cells underwent a one-week recovery period in a drug-free medium to mitigate acute cytotoxic effects and enable cellular recovery. After the recovery period, the cells were subjected to a second phase of treatment, consisting of three doses of cisplatin at the IC50 concentration (2.89 µM), again administered three days apart. Persister cells that survived the treatment were considered cisplatin-tolerant and were subsequently cultured with a constant presence of cisplatin at the IC25 concentration (IC25 of wt FaDu cells) for the entire experimental period to maintain the tolerant phenotype.

### 2.2. Cell Viability Assay

To assess the impact of HON and MAG on cell viability, the resazurin assay was performed. Cells (6 × 10^3^/well) were seeded onto black 96-well plates. The following day, fresh medium containing HON or MAG (at the concentration range of 10–40 µM) was added. The control cells were treated with the vehicle (DMSO). After 48 h of incubation, cells were washed with pre-warmed PBS buffer. Subsequently, resazurin (Sigma-Aldrich, St. Louis, MO, USA) solution was added to wells, and cells were incubated for an additional 90 min. Fluorescence (excitation λ = 530 nm, emission λ = 590 nm) was measured using the Infinite M200 multiplate reader (Tecan, Grödig, Austria). Each experiment was conducted independently at least three times, with at least six replicates per variant each time.

### 2.3. The Analysis of Cell Viability in 3D Culture

The cells (6 × 10^3^/well) were seeded into wells of ultra-low attachment 96-well plates (Corning, NY, USA). After the initial four days necessary for spheroid formation and growth, fresh medium containing the studied compounds was added into wells. Cell viability and cell death were measured after 72 h using the Cyto3D Live-Dead Assay kit (The Well Bioscience, North Brunswick, NJ, USA), according to the manufacturer’s protocol. The assay was based on the use of two nucleus staining dyes—acridine orange (AO, leading to green fluorescence reflecting the presence of live cells) and propidium iodide (PI, leading to red fluorescence reflecting the presence of dead cells). Images of spheroids in the bright field and stained with AO were taken using the JuliFL microscope (NanoEntek, Seoul, Republic of Korea). The experiment was repeated twice, with five replicates each time. 

### 2.4. The Analysis of the Cell Cycle

The analysis of the cell cycle was performed using Muse^®^ Cell Cycle Kit (Luminex, Austin, TX, USA) according to the manufacturer’s recommendations. Briefly, 1 × 10^5^/well of cells were seeded in a 12-well plate and pre-incubated for 24 h in a complete culture medium. Afterward, fresh medium containing the tested compounds was added, and cells were incubated for an additional 48 h. Cells treated with topotecan were used as a positive control of cell cycle arrest. Then, cells were collected by trypsinization, washed with PBS buffer, fixed in 70% ethanol, and stored at −20 °C overnight. For analysis, cells were stained with propidium iodide solution in the presence of RNase A, and the fluorescence of cells was analyzed with Muse Cell Analyzer (Merck, Darmstadt, Germany) after 30 min incubation in the dark at room temperature. Data analysis was performed using Muse 1.5 Analysis software (Merck, Darmstadt, Germany). The experiment was repeated three times.

### 2.5. The Evaluation of Apoptosis

The induction of apoptosis was analyzed using Muse Annexin V & Dead Cell Kit (Luminex, Austin, TX, USA) according to the manufacturer’s recommendations. Briefly, 1 × 10^5^/well of cells were seeded in a 12-well plate and pre-incubated for 24 h in a complete culture medium. Afterward, fresh medium containing the tested compounds was added, and cells were incubated for an additional 48 h. Cells treated with topotecan were used as a positive control of apoptosis induction. Then, cells were collected by trypsinization, and after centrifugation, the cell pellet was re-suspended in complete culture medium containing Annexin V for phosphatidylserine staining and 7-amino actinomycin D (7-AAD) for the detection of dead cells, in order to discriminate early and late apoptotic cells. After 20 min incubation in the dark at room temperature, flow cytometric assessment was performed with the Muse Cell Analyzer (Merck, Darmstadt, Germany). Data analysis was conducted using Muse 1.5 Analysis software (Merck, Darmstadt, Germany). The experiment was repeated three times.

### 2.6. The Evaluation of Gene Expression

The effect of the compounds on the expression of genes associated with the regulation of the cell cycle and apoptosis (*BAX*, *BIRC5*, *CCND1*, *CDKN1A*) was performed as previously described [31]. Briefly, FaDu cells (wild-type and cisplatin persister cells) were exposed to the compounds for 48 h, and RNA was extracted from cells using RNA Extracol (EURx, Gdańsk, Poland). The experiment was repeated twice. Reverse transcription was performed using smART First Strand cDNA Synthesis Kit (EURx, Gdańsk, Poland), according to the manufacturer’s protocol. Amplification was performed using SG qPCR Master Mix (EURx, Gdańsk, Poland) and the previously described gene-specific starters [31]. The mean expression of two reference genes (*PBGD*, *TBP*) was used to calculate the relative change in gene expression level (fold–change) in comparison to DMSO-treated cells.

### 2.7. Statistical Analysis

The statistically significant differences (*p* ≤ 0.05) between experimental and control groups were detected using Student’s *t*-test (GraphPad.com, accessed on 30 June 2024).

## 3. Results

### 3.1. FaDu Cisplatin Persister Cells Are More Sensitive to Viability Reduction by HON and MAG than FaDu wt Cells

**Figure 2:**
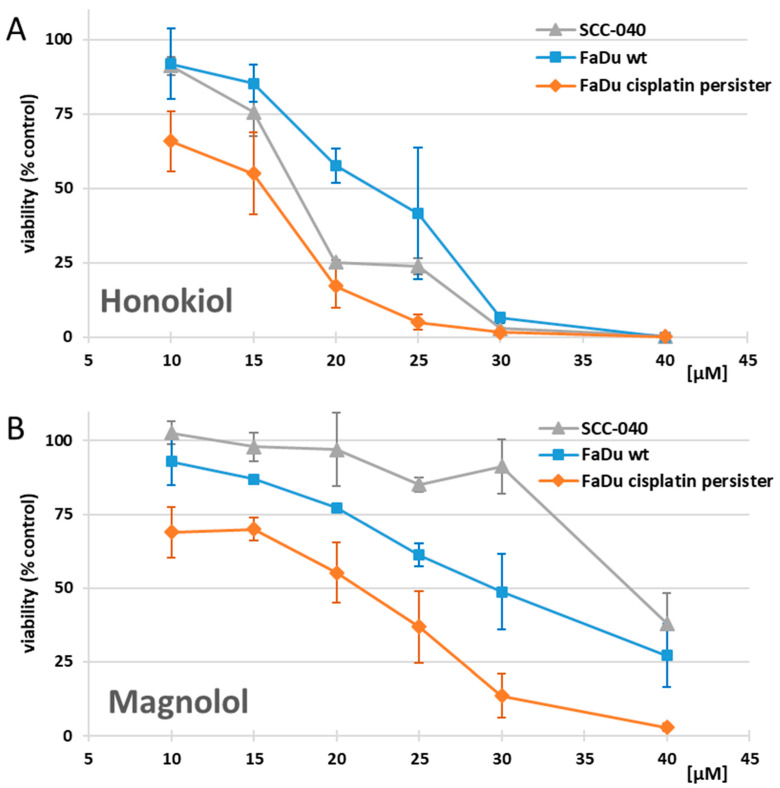
The effect of (**A**) honokiol and (**B**) magnolol on SCC-040, FaDu wt, and FaDu cisplatin persister cells viability based on the resazurin assay. Cells were treated with increasing concentrations of the compounds for 48 h. The results represent mean values ± SD from at least three independent experiments. Cells treated with the vehicle only were used as the control (100% viability).

Initially, we performed cell viability analyses to assess the anti-cancer properties of HON and MAG. In the 10–25 µM concentration range of HON, the SCC-040 cells were more affected than FaDu wt cells (Figure 2A). However, the activity of HON was similar at 30 µM and 40 µM concentrations, where both cell lines were almost totally affected. In turn, FaDu wt cells were more sensitive to MAG (Figure 2B). In this case, the viability curves showed weaker effects than for HON in both cell lines and reached approximately 73% and 62% reduction at 40 µM in FaDu wt and SCC-040 cells, respectively.

In addition, we established FaDu cisplatin persister cells to evaluate the potential activity of HON and MAG toward cisplatin-tolerant cells. HON and MAG reduced the viability of FaDu cisplatin persister cells more remarkably compared to FaDu wt cells. At the highest concentration used, the viability was close to 0%.

### 3.2. HON Reduces the Viability of HNSCC Cells Growing in Spheroid Form 

**Figure 3:**
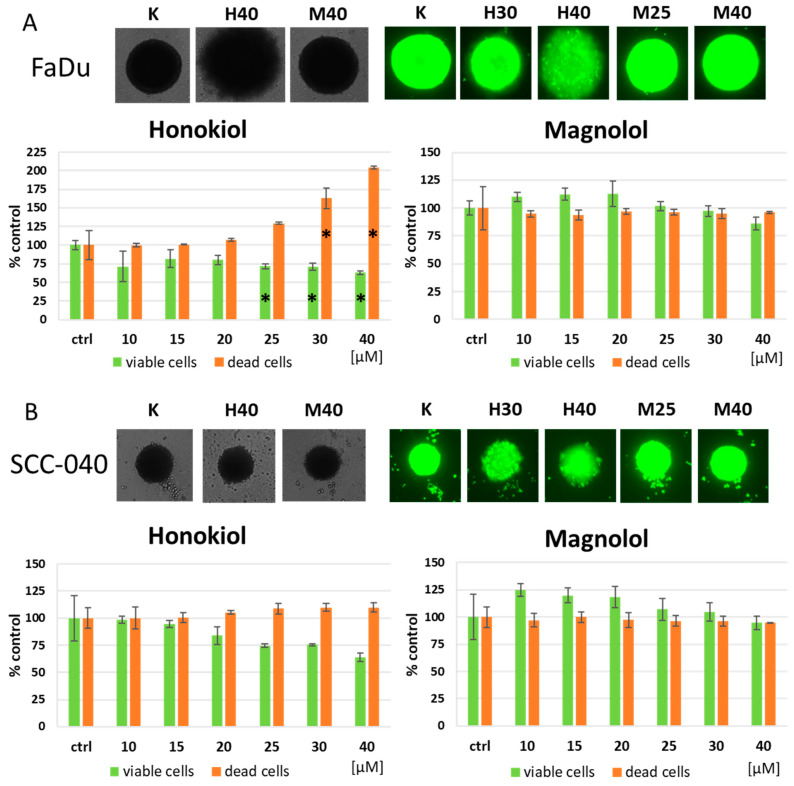
The effect of honokiol and magnolol on the viability of (**A**) FaDu and (**B**) SCC-040 cells grown in the form of spheroids. Cells were treated with increasing concentrations of the compounds for 72 h. Exemplary bright field images and images of stained cells (green fluorescence reflecting the presence of acridine orange in the nucleus of viable cells) are shown. The results in the graphs represent mean values ± SD from two experiments with five independent replicates each. The asterisk (*) denotes statistically significant differences in comparison to DMSO control (ctrl), *p* ≤ 0.05.

In the next step, we wanted to assess whether HON and MAG could induce anti-cancer effects in the spheroids of FaDu and SCC-040 cells, which constitute a better model for predicting in vivo activity. The results, which show the detection of both viable and dead cells, confirmed the potency of HON against FaDu cells (Figure 3A). As presented in the exemplary microscopic images, HON caused the collapse of the compact spheroid structure at the highest concentration. In turn, MAG had no significant impact on FaDu cell spheroids. In the 3D culture of SCC-040 cells, HON reduced the percentage of viable cells at higher concentrations, but the effects did not reach statistical significance (Figure 3B). Moreover, HON did not affect the proportion of dead cells. However, the destabilization of spheroids structure was observed at higher concentrations. SCC-040 cell spheroids were resistant to MAG, contrary to 2D results, where MAG at 40 µM reduced the viability below 50%.

Based on 2D and 3D viability analyses, it was shown that the activity of HON and MAG was more potent in FaDu cells. Moreover, FaDu cisplatin persister cells were more affected by these natural compounds. Thus, we performed additional functional and mechanistic analyses using wild-type and cisplatin persister FaDu cells to further characterize the action of HON and MAG. 

### 3.3. HON and MAG Induce Cell Cycle Arrest in G1/G0 Phases in FaDu Cells

**Figure 4:**
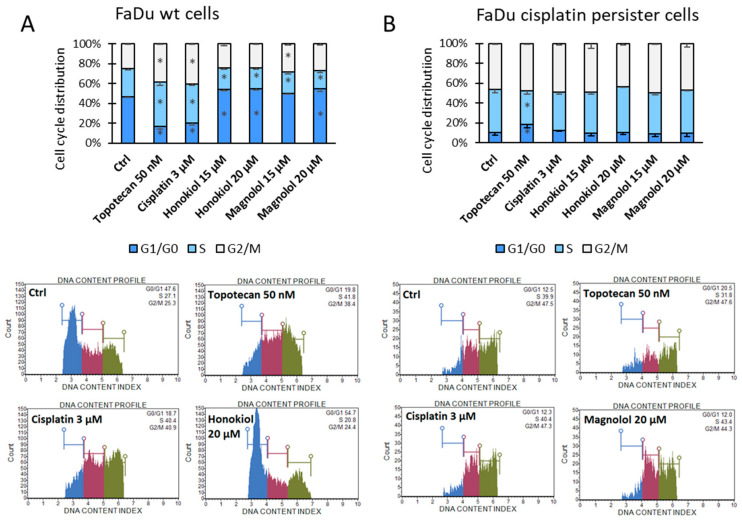
Cell cycle distribution analysis after propidium iodide (PI) staining in (**A**) FaDu wild-type (wt) cells and (**B**) FaDu cisplatin persister cells. The cells were incubated with cisplatin, honokiol, or magnolol for 48 h. Topotecan was used as the positive control of cell cycle arrest. Exemplary flow cytometry plots are shown. The blue area denotes the G1/G0 phases, the red area denotes the S phase, and the green area denotes the G2/M phases of the cell cycle. The results represent mean values ± SD from three independent experiments. The asterisk (*) denotes statistically significant differences in comparison to DMSO control (Ctrl) for each cell cycle phase, *p* ≤ 0.05.

The cell cycle analysis was introduced to evaluate the possible differences in HON and MAG activity between FaDu wt and FaDu cisplatin persister cells (Figure 4). In FaDu wt cells, the typical change observed was the cell cycle arrest in G1/G0 phases with a concomitant reduction in cell percentage in the S phase. Only with MAG at 15 µM concentration a slight enrichment of G2/M cell cycle phases was observed instead. In turn, cisplatin, along with the positive control, topotecan, presented a potent cell cycle arrest in the S and G2/M phases (Figure 4A).

In FaDu cisplatin persister cells, due to the lengthy exposure of cells to cisplatin, the cell cycle distribution was similar to the effect of cisplatin in wt FaDu cells, with an even smaller percentage of cells in G1/G0 phases (Figure 4B). HON and MAG were unable to trigger additional effects. In these cells, only topotecan was able to change the proportion between the G1/G0 and S phases.

### 3.4. HON and MAG Are Weaker Apoptosis Inducers than Cisplatin in FaDu Cells

**Figure 5:**
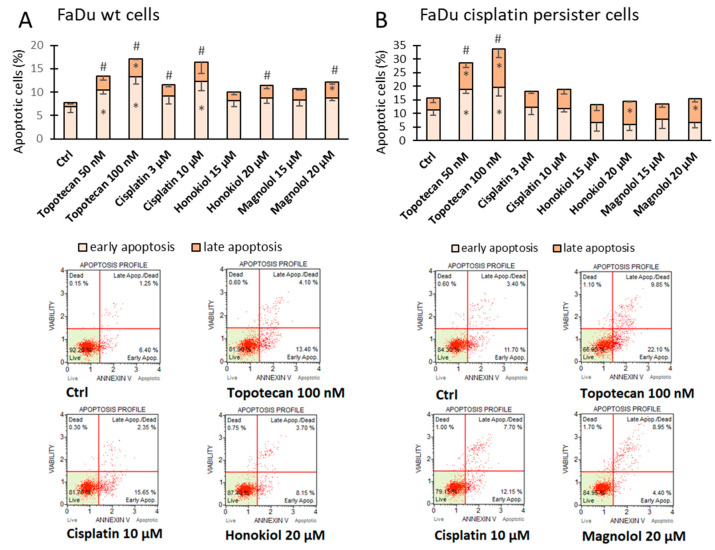
Apoptosis induction analysis after Annexin V and 7-amino actinomycin D staining in (**A**) FaDu wild-type (wt) cells and (**B**) FaDu cisplatin persister cells. The cells were incubated with cisplatin, honokiol, or magnolol for 48 h. Topotecan was used as the positive control. Exemplary flow cytometry plots are shown. The results represent mean values ± SD from three independent experiments. Statistically significant differences in comparison to DMSO control (Ctrl) for early and late apoptosis are represented by an asterisk (*), while for total apoptotic cells, the hash (#) was used, *p* ≤ 0.05.

Annexin V-based flow cytometry analysis revealed a concentration-dependent increase in total apoptotic FaDu wt cells for topotecan (positive control) and cisplatin (Figure 5A). The results for HON and MAG were similar, and an approximately 50% increase in total apoptotic cell population was found for the chemicals at 20 µM concentration. In the case of MAG, an increase in the rate of late apoptotic cells was also present.

In FaDu cisplatin persister cells, an increase in total apoptotic cell population was shown only in topotecan-treated cells (Figure 5B). The lack of effects after treatment of cells with cisplatin confirmed their cisplatin persister status. Interestingly, although there was no enrichment in total apoptotic rate after incubation of cells with HON or MAG, the proportions between early and late apoptosis were modified with a doubled amount of late apoptotic cells for the chemicals at 20 µM concentration. 

### 3.5. HON and MAG Act by Altering the Level of Expression of BIRC5 and CDKN1A Genes

**Figure 6:**
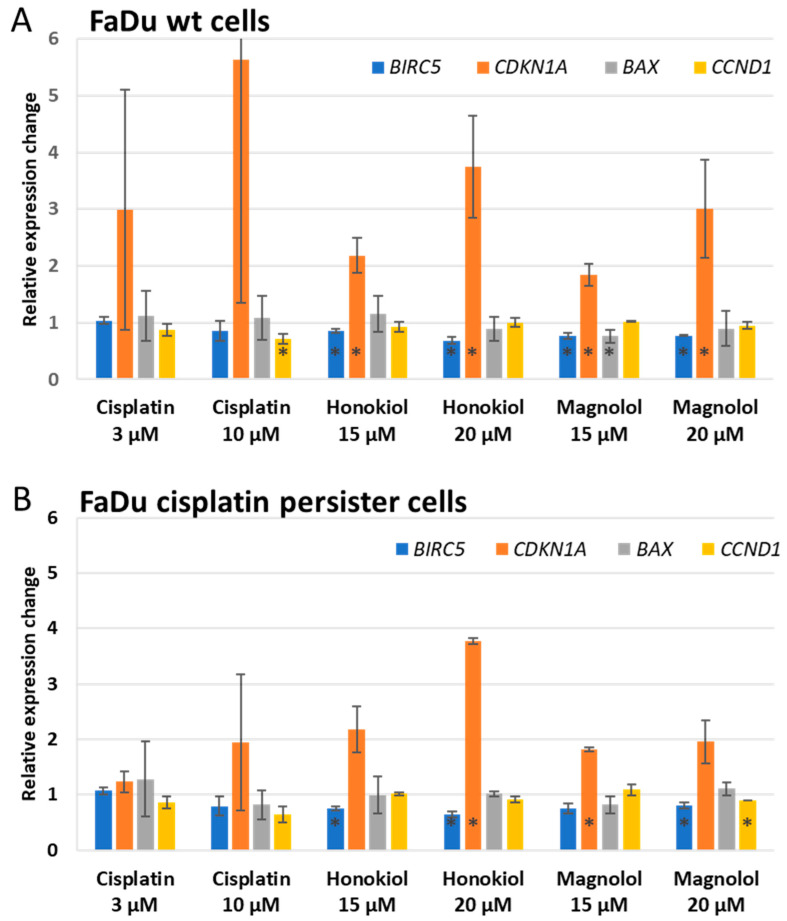
The results of the analysis of the changes in the level of expression of *BIRC5*, *CDKN1A*, *BAX*, and *CCND1* genes in (**A**) Fadu wt and (**B**) FaDu cisplatin persister cells. The cells were incubated with cisplatin, honokiol, or magnolol for 48 h. The results represent fold–change mean values ± SD from two independent experiments. The asterisk (*) inside bars denotes statistically significant differences in comparison to DMSO control, *p* ≤ 0.05.

In an attempt to explain the mechanism of action of HON and MAG in FaDu cells, we evaluated their effect on the level of expression of genes associated with the regulation of cell cycle and apoptosis, and we compared it to the effect exerted by cisplatin. In general, the effect of cisplatin was related to the tendency to decrease the level of expression of *CCND1* (which encodes the cell cycle stimulatory cyclin D1) and to increase the expression of *CDKN1A* (which encodes the cell cycle inhibitory p21 protein) in a dose-dependent manner in both cell populations, although the change reached statistical significance only in the case of *CCND1* reduction in FaDu wt cells at 10 µM concentration (Figure 6). On the other hand, HON and MAG acted by decreasing the level of expression of *BIRC5* (which encodes the pro-survival protein survivin) and by increasing the expression of *CDKN1A*, which was more pronounced in FaDu wt cells.

## 4. Discussion

Magnolia tree is a source of two compounds, honokiol, and magnolol, which have a pleiotropic activity that supports human health. In this study, we particularly focused on HON and MAG anti-cancer properties. Thus far, their potency against cancer cell growth has been evaluated in many tumor types, including colon, breast, liver, lung, and brain cancer, to name a few [21,32]. On the other hand, research on HNSCC has mainly concerned HON, and the influence of MAG has been poorly described so far.

Initially, we analyzed the influence of HON and MAG on the viability of HNSCC cells. It was shown that HON in concentrations reaching 40 µM demonstrated more substantial effects against SCC-040, FaDu wt, and FaDu cisplatin persister cells than its structural isomer MAG. A total of 30 µM HON reduced the viability below 10% of the control value, and 40 µM HON was entirely toxic. Based on other studies, the activity of HON in HNSCC is cell line-dependent. On the one hand, HON within the concentration range of 30–40 µM similarly affected OC2 and OCSL cell lines [25] or the HN-22 cell line at the concentration of 10 µg/mL (approximately 38 µM) [33]. On the other hand, the viability of several other HNSCC cells was significantly inhibited only at higher concentrations, e.g., HSC-3 and HSC-4 cell lines [28,34] or SCC-1 and SCC-5 cell lines [35]. Concerning MAG, our results confirmed its dose-dependent potential to diminish the viability of FaDu cells, with almost 75% reduction in the highest 40 µM concentration. In turn, SCC-040 cells were not meaningfully affected within the range of 10–30 µM MAG, but at 40 µM, the viability decreased below 50%. Other available data indicated even weaker activity of MAG in HSC-3 and SCC-9 cell lines, where at least 75 µM concentration was needed to achieve a decrease in cell viability below 50% [36]. 

In addition to 2D cell cultures, we also analyzed the influence of HON and MAG on the viability of FaDu and SCC-040 cells grown in 3D in the form of spheroids. Fluorescence analysis with concomitant microscopic observation of spheroids confirmed the anti-cancer activity of HON. However, the activity of MAG in HNSCC cells grown in 3D form was not potent enough. Therefore, based on viability analyses, HON presented more beneficial anti-cancer effects. The reason could be related to the mechanism of action of HON. For instance, Singh et al. (2015) [35] detected decreased EGFR, mTOR, and downstream targets expression after the exposure of HNSCC cells and their xenografts in athymic nude mice to HON. Moreover, molecular docking analysis proved the ability of HON to bind the EGFR active site with greater potency than the commonly used EGFR tyrosine-kinase (TK) domain inhibitor gefitinib. In our previous research, erlotinib, another EGFR TK domain inhibitor, reduced the viability of CAL 27 and FaDu cell spheroids even better than in 2D models or compounds with different mechanisms of action [37]. Therefore, HNSCC cell spheroids are possibly a good model for detecting sensitivity to anti-EGFR therapy, and from the two analyzed *Magnolia*-derived compounds, only HON affects EGFR signaling. In xenografts of HNSCC cells, a combination of HON with erlotinib [38] and HON with cetuximab (monoclonal antibody against the extracellular domain of EGFR) [29] caused a more significant reduction in tumor volume in comparison to those chemicals used individually. Thus, HON is promising as an adjuvant for treatments targeting EGFR in HNSCC cells, but other HON modes of action should be taken into account as well.

In this study, we also compared the sensitivity of FaDu wt and FaDu cisplatin persister cells to HON and MAG. Both compounds demonstrated a more pronounced decrease in viability in cisplatin persister cells, which may suggest the appearance of sensitization of FaDu cisplatin persister cells to HON and MAG. Therefore, in further experimental steps, we used these two populations of FaDu cells to assess the influence of HON and MAG on the cell cycle distribution and potential induction of apoptosis. We chose the same concentrations for HON and MAG (15 µM and 20 µM) to compare their activity and keep the concentration within values possible to reach in vivo [39]. In addition, cisplatin was tested in parallel as the reference of cellular response to standard chemotherapy. 

Changes in the percentage of cells in each cell cycle phase were observed in FaDu wt cells. Cisplatin caused a typical increase in S and G2/M phases related to its mechanism of action [40]. In turn, HON and MAG provoked an increase in the G1 phase. This result is in line with the reports of other researchers and is related to decreased proliferation of cancer cells. Particularly, G1 cell cycle arrest was observed in OC2 and OCSL cell lines for HON [25] and HSC-3 and SCC-9 cell lines for MAG [36]. The flow cytometric analysis of apoptosis revealed an approximately 50% increase in apoptotic cell population as compared to control by HON and MAG in FaDu wt cells, while cisplatin doubled the apoptotic rate. The results of our study suggest that FaDu wt cells can be considered sensitive to the pro-apoptotic effect of HON and MAG applied at lower concentrations (≤20 µM). In contrast, the induction of apoptosis by HON was detected at significantly higher concentrations, 40 µM and 30 µM, in OC2 and OCSL cell lines, respectively [25]. For HSC-3 and HSC-4 cell lines, similar potency was shown for 10 µg/mL (ca. 38 µM) concentration of HON and extensive apoptosis for 15 µg/mL (ca. 56 µM) and 20 µg/mL (ca. 75 µM) [34]. Also, 75 µM MAG was needed to induce apoptosis in HSC-3 and SCC-9 cell lines [36].

FaDu cisplatin persister cells, due to lengthy exposure to cisplatin, were characterized by changes in the cell cycle distribution, similar to the effect of 48 h incubation of FaDu wt cells with cisplatin. Thus, the additional incubation of persister cells with cisplatin did not further change the distribution of cells across phases. HON and MAG did not exert any effects either. The basal apoptotic rate in FaDu cisplatin persister cells was higher than in FaDu wt cells. Nevertheless, topotecan (positive control) could still lead to a significant increase in the apoptotic cell population. Importantly, cisplatin had no significant impact on apoptosis, confirming the resistance of these persister cells to cisplatin. In parallel, HON and MAG lacked the potency to increase the rate of apoptotic cells. Nevertheless, HON and MAG doubled the percentage of late apoptotic cells, which may suggest a partly different mechanism of HON and MAG activity in FaDu cisplatin persister cells.

In order to assess the possible mechanisms responsible for the observed effects, we evaluated the level of expression of genes related to cell cycle and apoptosis control. The impact of HON and MAG on the expression of anti-apoptotic and pro-proliferative genes supports the observations from cytometric analyses. Survivin (encoded by the *BIRC5* gene) is a member of the inhibitor of apoptosis (IAP) family but can also promote cell cycle progression. Another protein, p21 (*CDKN1A*), is promoted by p53 to inhibit cell cycle progression. In HN-22 and HSC-4 cell lines, HON decreased the expression of survivin and increased the level of p21, which was related to the downregulation of the transcription factor specificity protein 1 (Sp1) [33]. Our results for FaDu wt and FaDu cisplatin persister cells confirm this observation. Another pro-apoptotic gene, *BAX,* was affected only by MAG 15 µM in FaDu wt cells, while the expression of cyclin D1 (*CCND1*), which promotes cell cycle progression, was slightly downregulated by MAG 20 µM in FaDu cisplatin persister cells. Survivin is known as an adverse prognostic factor for HNSCC patients and is involved in DNA damage repair induced by radiotherapy. HON improved the effects of radiotherapy by targeting survivin, as shown in the in vitro and xenograft models of HNSCC [41]. Therefore, the lowered expression of survivin upon treatment with HON and MAG highlights their usefulness as anti-cancer compounds. 

Our study confirmed the anti-cancer activity of HON and MAG in HNSCC cells. The compounds reduced the viability of SCC-040, FaDu wt, and FaDu cisplatin persister cells. Moreover, FaDu cisplatin persister cells were the most susceptible to the influence of HON and MAG on viability. The potency of HON and MAG in assays evaluating their effect on cell cycle and apoptosis was similar; however, slight differences in the ability to modulate the expression of genes related to those processes were found. On the other hand, the anti-cancer effects against HNSCC cells observed in this study appeared at relatively high concentrations, which may limit the in vivo effectiveness of HON and MAG. Yet, while this study was limited to in vitro experiments, reports from other researchers, including in vivo xenograft studies [25,26,28,29,35,38], indirectly confirm the beneficial activity of HON and MAG in treating HNSCC. Moreover, these natural-derived compounds can also be valuable for the prevention of oral cancer development. In this regard, fibrotic buccal mucosal fibroblasts (fBMF) were much more affected by HON than normal BMF, and the progression of oral fibrogenesis into cancer was inhibited [42]. Interestingly, the safety profile of HON is also beneficial. HON can even prevent cisplatin ototoxicity without compromising its activity against tumor cells of different origins [43]. Yet, while showing a generally favorable safety profile, HON and MAG may still cause significant side effects, including hemorrhage due to the anti-thrombotic activity. In addition, they have been shown to affect the activity of enzymatic systems responsible for glucuronidation or sulfation reactions, which may lead to altered drug metabolism [44]. Thus, further research on the anti-cancer properties of HON and MAG is necessary.

## 5. Conclusions

This study confirmed that honokiol and magnolol can limit head and neck cancer cell viability. These effects are contributed to by the regulation of the cell cycle and apoptosis, which may be mechanistically related to alterations in the level of expression of *BIRC5* (encoding survivin) and *CDKN1A* (encoding p21) induced by HON and MAG. Although HON and MAG showed much higher potency against wild-type FaDu cells, the chemicals were able to reduce the viability and increase the late apoptosis rate in FaDu cisplatin-persister cells. This implies the potential applicability of HON and MAG in tackling chemoresistance, although this requires further, more detailed studies.

## Data Availability

The data generated in the present study are available from the corresponding author upon reasonable request.
